# Null Effects on Working Memory and Verbal Fluency Tasks When Applying Anodal tDCS to the Inferior Frontal Gyrus of Healthy Participants

**DOI:** 10.3389/fnins.2018.00166

**Published:** 2018-03-19

**Authors:** Samuel J. Westwood, Cristina Romani

**Affiliations:** School of Life & Health Sciences, Aston University, Birmingham, United Kingdom

**Keywords:** null effects, tDCS, verbal fluency, working memory, brain stimulation

## Abstract

Transcranial direct current stimulation (tDCS) is a technique used to modify cognition by modulating underlying cortical excitability via weak electric current applied through the scalp. Although many studies have reported positive effects with tDCS, a number of recent studies highlight that tDCS effects can be small and difficult to reproduce. This is especially the case when attempting to modulate performance using single applications of tDCS in healthy participants. Possible reasons may be that optimal stimulation parameters have yet to be identified, and that individual variation in cortical activity and/or level of ability confound outcomes. To address these points, we carried out a series of experiments in which we attempted to modulate performance in fluency and working memory probe tasks using stimulation parameters which have been associated with positive outcomes: we targeted the left inferior frontal gyrus (LIFG) and compared performance when applying a 1.5 mA anodal current for 25 min and with sham stimulation. There is evidence that LIFG plays a role in these tasks and previous studies have found positive effects of stimulation. We also compared our experimental group (*N* = 19–20) with a control group receiving no stimulation (*n* = 24). More importantly, we also considered effects on subgroups subdivided according to memory span as well as to more direct measures of executive function abilities and motivational levels. We found no systematic effect of stimulation. Our findings are in line with a growing body of evidence that tDCS produces unreliable effects. We acknowledge that our findings speak to the conditions we investigated, and that alternative protocols (e.g., multiple sessions, clinical samples, and different stimulation polarities) may be more effective. We encourage further research to explore optimal conditions for tDCS efficacy, given the potential benefits that this technique poses for understanding and enhancing cognition.

## Introduction

Transcranial direct current stimulation (or tDCS) is a non-invasive form of brain stimulation which is used to modulate cognitive performance by applying a weak electric current via electrodes placed on the scalp. Early studies measuring effects of tDCS on motor cortical excitability suggested that the applied current can cause directional changes in the resting membrane potentials underneath the electrodes—with predominant depolarization under the anode (known as anodal tDCS) vs. hyperpolarization under the cathode (cathodal tDCS; de Berker et al., 2013). It is widely assumed that effects on cortical excitability map on to cognitive effects, with anodal vs. cathodal tDCS improving vs. worsening the cognitive function of targeted brains regions. However, though widely assumed, this might not necessarily be the case. Current flows between the electrodes with complex effects that are poorly understood. Moreover, an important confounding factor modulating the impact of tDCS may be individual variation in cortical activity and/or level of ability (for reviews, see Miniussi et al., 2013; Horvath et al., 2015; Li et al., 2015; Westwood and Romani, 2017; Westwood et al., 2017). These are widely cited as explanations for a number of recent reports of negative, inconsistent, and/or small effects linked to single applications of tDCS especially in healthy participants (see Horvath et al., 2015; Mancuso et al., 2016; Westwood et al., 2017). Our study will contribute to clarify the scope of tDCS effects by considering tasks that tax executive selection abilities, mediated by the frontal lobes, and where positive, but inconsistent, effects have been reported before. We will consider effects on the whole participant group, but crucially also on subgroups subdivided according to (a) general performance and control abilities; (b) working memory span; and (c) motivation levels to see whether these variables affect tDCS outcomes.

We will tap executive selection using verbal fluency tasks and probe tasks. In fluency tasks, participants have to name in 60 s as many unique words as possible that belong to a given semantic category (semantic fluency) or begin with a given letter (phonemic fluency; for review, see Whiteside et al., 2016). In probe tasks, participants judge yes or no whether a test item (or probe) was present in a target list presented immediately before (e.g., referred to as *Sternberg task*; *recent-probe*; *Deese-Roediger-McDermot*, or *DRM*) or in a particular position in a continuous sequence of items (e.g., the n-back task; for reviews, see Jonides and Nee, 2006; Irlbacher et al., 2014). We will attempt to modulate executive selection on these tasks by targeting the left inferior frontal gyrus (LIFG) with anodal tDCS. Various lines of research suggest that this brain region plays an important role in supporting performance on these tasks, and in executive selection processes more generally (see Hirshorn and Thompson-Schill, 2006; Jonides and Nee, 2006; Badre, 2008; Nelson et al., 2009; Atkins et al., 2011; Robinson et al., 2012; Biesbroek et al., 2016), with positive effects reported with tDCS and other forms of non-invasive brain stimulation (Feredoes et al., 2006; Price et al., 2015; Hill et al., 2016).

One expectation is that we will find a beneficial effect of anodal tDCS on task performance, but that possibly this effect will not be uniform across participants. Optimal executive selection is a dynamic interplay between automatic activation and controlled modulation of this activation—e.g., some activated responses will be selected whilst others are suppressed in the service of a goal (Thompson-Schill et al., 2005; Barak and Tsodyks, 2014; Sprekeler, 2017). Individual differences in the capacity to recruit control mechanisms will potentially interact with the tDCS effect resulting in either a net positive or negative outcome (Krause et al., 2013; Krause and Kadosh, 2014). For example, one possibility is that if selection is operating at optimum levels at baseline, anodal tDCS may have no effect or may increase excitability beyond the optimum working range, but, if selection is poor at baseline then tDCS may boost this ability (as well as increase general activation levels) with net positive outcomes. This is consistent with evidence that effects of anodal tDCS are determined by working memory span (see Berryhill and Jones, 2012; Jones and Berryhill, 2012; Berryhill et al., 2014; Jones et al., 2015; Gözenman and Berryhill, 2016), and baseline levels of inhibitory control and task ability (Sela et al., 2012; Tseng et al., 2012; Hsu et al., 2014, 2016; Jones et al., 2015; London and Slagter, 2015). The impact of such individual differences will additionally be compounded by task mediated demands on executive selection processes.

We chose to target fluency and probe tasks because they are particularly apt for exploring task mediated variation in executive selection. In verbal fluency, some areas of the lexicon will be activated, but participants will have to carefully match items to selection criteria, whilst inhibiting earlier responses. More importantly, because participants prefer to produce clusters of words similar in meaning (e.g., dog, cat, mouse) and/or sound (e.g., lift, link, listen), exhausted clusters need to be inhibited whilst a new selection criterion is generated in order to switch to a new cluster (see Shao et al., 2014; Berberian et al., 2016; Whiteside et al., 2016). In probe tasks, one can devise conditions that introduce lure probes that are either related (e.g., semantically or associatively) to items in the target list (e.g., the *DRM* task or a variant used in this study, the *semantic-associated probe*), were presented in a previous list (e.g., *recent-probe*), or—in the case of the n-back task—one can place targets next to the target position in the sequence (e.g., placing the target 3-back in a 2-back task, such as the target j in the sequence j, a, b, j in a 2-back task; see seminal work by Gray et al., 2003). In both cases, control resources must be deployed to update contents in working memory and to suppress lures which will otherwise bias responses due to their relatedness or familiarity with list items (Jonides and Nee, 2006; Novick et al., 2010; Atkins et al., 2011; Irlbacher et al., 2014).

One key aim of this investigation is to subdivide participants on a number of measures to see whether differences on these measures can predict differences in response to tDCS. Firstly, we will use general measures of performance in terms of overall performance on verbal fluency and probe tasks and digit span. Secondly, we will use more direct measures of executive control. For the fluency tasks, we will divide participants based on the number of correct switches over the total number of correct responses at baseline; the assumption being that—in line with previous studies—greater switching reflects better control abilities (see Troyer et al., 1998; Hirshorn and Thompson-Schill, 2006). For the probe tasks, we will consider the difference in performance between lists containing neutral vs. lure probes, the assumption being that a smaller interference from lures reflects better control abilities (see Hirshorn and Thompson-Schill, 2006; Jonides and Nee, 2006; Irlbacher et al., 2014; Shao et al., 2014). Finally, we will use a measure of motivation to succeed on a task because it has been shown previously that participants who score higher in this trait perform better on working memory tasks (for review, see Fino et al., 2014) and are more amenable to tDCS modulation (see Metuki et al., 2012; Sela et al., 2012; Jones et al., 2015). For this we will use the BAS component of BIS/BAS scale (Behavioral Approach System/Behavioral Inhibition System, Carver and White, 1994), which measures the reward sensitivity trait (for similar method, see Metuki et al., 2012; Sela et al., 2012), and is correlated positively with working memory and cognitive control abilities more generally (Gray and Braver, 2002; for reviews, see Gray and Burgess, 2004; Savine et al., 2010; Fino et al., 2014).

Before moving to our experimental investigation, we will now briefly review existing studies assessing the effects of tDCS on verbal fluency and probe tasks.

For *verbal fluency*, early reports found promising evidence that applying anodal tDCS to the left prefrontal cortex for up to 20 min can increase the average number of words produced (Iyer et al., 2005; Cattaneo et al., 2011). However, not all studies reported positive results (see Cerruti and Schlaug, 2009; Vannorsdall et al., 2012, 2016; Penolazzi et al., 2013a; Binney et al., 2018). In a meta-analysis, Price et al. (2015) found small to moderate effects (roughly 0.5, Hedges' *g*) for anodal tDCS in studies measuring verbal fluency (*n* = 6) or language learning (*n* = 2) when pooling all studies together. Positive effects were also found for studies measuring offline effects on verbal fluency (*n* = 3). However, significant effects were potentially carried by three effect size estimates, which were exceptionally large relative to others (0.8 = Flöel et al., 2008; 1.1 = Cattaneo et al., 2011; 0.7 = Meinzer et al., 2012). Studies generally find more significant effects of anodal tDCS with semantic compared to phonemic fluency (see Cattaneo et al., 2011; Horvath et al., 2015; Price et al., 2015). Only three studies have measured clustering and switching. Two applied anodal tDCS to left frontal regions, with one study showing an increase in cluster sizes in semantic fluency (Vannorsdall et al., 2012), whist another showed no effect at all (Penolazzi et al., 2013b). The third study targeted dorsal-frontal, temporal-parietal, and frontal-temporal regions, and found that only cathodal tDCS applied to the frontal-temporal regions increased cluster sizes (see Binney et al., 2018). However, unlike previously mentioned studies, this last study used a three electrode montage which limits comparisons (e.g., two cathodes placed bilaterally over the left *and* right hemisphere).

For *working memory*, a number of reviews report similarly mixed results. One review reported anodal tDCS related gains on both reaction times and accuracy scores (see Hill et al., 2016), another only on reaction times (see Brunoni and Vanderhasselt, 2014). A more comprehensive review found small to null effects across reaction times and accuracy scores following their own meta-analysis and a re-analysis of the two previous meta-analyses (Mancuso et al., 2016). Instead, a significant but small effect was seen for working memory training (Mancuso et al., 2016). However, a majority of tDCS studies measuring effects on working memory do not directly measure performance during lures trials. Since lure trials place a greater load on hard to recruit control mechanisms, tDCS has more scope to modulate performance, as discussed previously. This may explain the small to null effects reported in the above meta-analysis, which pool predominantly from studies using n-back tasks without lures, and the positive effects seen on probe tasks that include lures (such as the modified recent-probe task and semantic associated probe tasks).

Gladwin et al. (2012), for instance, reported that anodal tDCS to the *left dorsolateral prefrontal cortex* (dlPFC) decreased reaction times on lures trials in their modified version of the *recent*-*probe* task, but no effect was found on neutral trials. By contrast, when using the *Sternberg* task (which does not include lures), two studies reported null effects (see Mulquiney et al., 2011; Teo et al., 2011), another found that cathodal but not anodal tDCS improved performance (e.g., Ferrucci et al., 2008), whilst oscillatory anodal *and* cathodal tDCS worsened performance in another study (Marshall et al., 2005). However, effects in this last study might be attributable to unconventional stimulation parameters (e.g., bifrontal tDCS, with intermittent stimulation see also Discussion). Positive effects on lure trials but not on non-lure trials were also reported on a modified n-back task, when applying High Definition (or HD) tDCS to the dlPFC. However, given that HD-tDCS uses a multi-electrode array that improves current focality, it is difficult to infer whether this positive effect was mediated by the presence of lures or stimulation parameters (see Hussey et al., 2015).

Other studies report similar effects targeting the *temporal* or *parietal* regions, two regions that support performance on probe tasks that include semantically and/or associatively related lures (for review, see Lambon et al., 2001; Jefferies and Lambon Ralph, 2006; Binder and Desai, 2011; Jefferies, 2013; Mirman and Britt, 2014). One study reported that anodal tDCS applied to left anterior temporal lobe can decrease false alarms for semantically related lures (Boggio et al., 2009), whilst another reported a decrease in false alarms for associative, but not semantically related lures (Díez et al., 2017). Other studies showed an increase in hits (i.e., correct *yes* responses to probes) when targeting the parietal cortex with a bilateral montage (i.e., right-anodal/left-cathodal), whilst the opposite montage (i.e., left-anodal/right cathodal) *increased* false alarms (see Pergolizzi and Chua, 2015, 2016; but see also, Pergolizzi and Chua, 2017).

## Aims of study

In our experimental investigation, we intend to stimulate the LIFG to evaluate factors that may drive the inconsistent effects seen in verbal fluency and probe tasks. In regards the latter, we chose to focus on recent-probe and semantic-associated probe, since studies using similar tasks have found positive effects of anodal tDCS, and because one can measure the differential impact of tDCS on control mechanisms during performance on non-lure and phonological and semantic lures in similar types of tasks. No study to our knowledge has applied anodal tDCS to the LIFG in these tasks, despite evidence from studies using other forms of non-invasive brain stimulation (Feredoes et al., 2006), and the role this region plays in switching and interference resolution on lure trials, and in executive selection more generally (for reviews, see Jonides and Nee, 2006; Badre and Wagner, 2007; Badre, 2008). We will measure performance across the whole group of participants, but also subdivide participants based on different measures which may modulate the effect of tDCS. We hypothesize that anodal tDCS would improve performance across tasks, but this may change in accordance with individual and task mediated variation in executive selection, with a preferential effect on individuals with suboptimal executive selection abilities since these are more likely to be the beneficiaries of a boost potentially provided by tDCS.

## Experiments 1 and 2: tDCS effects on fluency and probe tasks

### Materials and methods

#### Design

All participants completed two testing sessions 1 week apart. Experimental participants carried out one session with active tDCS and one with sham tDCS. Control participants carried out two sessions without any form of stimulation. All participants carried out two parallel versions of the semantic and phonemic fluency tasks (*Experiment 1*) and two parallel versions of a *recent-probe* and a *semantic-associated probe* task *(Experiments 2*) across the two testing sessions. For the experimental participants, one group carried out the recent-probe whilst another group carried out the semantic-associated probe, but both groups carried out the phonemic *and* semantic fluency tasks. The control participants carried out all tasks. In the first session, the experimental participants were also administered a digit span task and the BIA/BAS scale before stimulation.

The time taken to complete one version of a given probe task and the verbal fluency tasks was roughly 20 min. We counterbalanced the order of session, stimulation, and task version across participants. To avoid participants using words presented in the probe task for their responses in the fluency tasks, fluency was always performed before the probe tasks.

#### Transcranial direct current stimulation (tDCS)

Stimulation was administered via a battery driven NeuroConn DC-Stimulator using a 25 cm^2^ anode and 35 cm^2^ cathode inserted in sponges soaked in saline solution. The anode was placed on the LIFG, whilst the cathode was placed on the contralateral supraorbital area. The LIFG was located as F7 in the 10/20 EEG system, which we located by measuring 2 cm from the corner of the eye to the ear then 3 cm at perpendicular upwards (see Gough et al., 2005). We administered a 1.5 mA current for 25 min. Stimulation was administered 5 min before participants performed the first (fluency) task, and continued throughout the duration of the other tasks (for the same method, see Westwood et al., 2017). These parameters were in line with previous studies (see Table 1). To assess the integrity of blinding, participants were asked about their experience of tDCS via a feedback questionnaire at the end of each session (see Fertonani et al., 2010).

**Table 1:** Table summarizing protocols used by previous studies measuring effects on verbal fluency and probe tasks including protocol used in present study at the bottom in bold.

**Author**	**A,C**	**Timing On/Off line**	**Target**	**Active cm^2^**	**mA**	**mA/cm^2^**	**Mins**	**Ref**	**Task**	**Sig?**
**VERBAL FLUENCY TASKS**										
Binney et al., 2018	A,C	Off	Fr-Te	5	2	0.2	20	Fpz	PF, SF	Y
	A,C	Off	Do-Fr	5	2	0.2	20	Fpz	PF, SF	N
	A,C	Off	Te-Pa	5	2	0.2	20	Iz	PF, SF	N
Cattaneo et al., 2011, Exp 1	A	Off	LIFG	35	2	0.06	20	CS	PF, SF	Y
Cattaneo et al., 2011, Exp 2	A	Off	RIFG	35	2	0.06	20	CS	PF, SF	N
Cerruti and Schlaug, 2009, Exp 1	A,C	Off	LdlPFC	16	1	0.06	20	CS	SF	N
Cerruti and Schlaug, 2009, Exp 2	A	Off	R/LdlPFC	16	1	0.06	20	CS	SF	N
Ehlis et al., 2016	A,C	Off	LIFG	35	1	0.03	20	CS	PF,SF	N
Martin et al., 2017	A	On	M1	35	1	0.03	30	CS/RM	SF	Y
Meinzer et al., 2012	A	On	LIFG+ATL	35	1	0.03	17	CS	SF	Y
Penolazzi et al., 2013a,b	A	Off(+20)	LIFG	35	2	0.06	20	CS	SF	Y
	A	Off(+20)	LIFG+ATL	35	2	0.06	20	CS	SF	N
	A	Off(+20)	LIFG+ATL	35	2	0.06	20	RH	SF	N
	A	Off(+20)	LIFG+ATL	35	2	0.06	20	CS	SF	N
Pisoni et al., 2017	A	On	LIFG	16	0.75	0.05	20	CS	PF, SF	Y
Vannorsdall et al., 2012	A	On	LdlPFC	25	1	0.04	30	V	PF, SF	N
Vannorsdall et al., 2016	A	Off	LIFG	35	2	0.06	20	CS	PF, SF	Y
**PROBE TASKS**										
Boggio et al., 2009	A	On/Off	LTC	35	2	0.06	10	RH	DRM	Y
Díez et al., 2017	A,C	On/Off	LATL	35	2	0.06	20	RS	DRM	Y
Ferrucci et al., 2008	A,C	On	RC	21	2	0.1	15	RD	S	Y
Ferrucci et al., 2008	A,C	On	LdlPFC	21	2	0.1	15	RD	S	Y
Gladwin et al., 2012	A	On/Off	LDLPFC	35	1	0.03	10	CS	MS	Y
Marshall et al., 2005	A,C	On	L/RdlPFC	0.8	0.26	0.33	15 s*	M	S	Y
Mulquiney et al., 2011	A	Online	LdlPFC	35	1	0.03	10	CS	S	N
Pergolizzi and Chua, 2016	A	Offline	LPC	35	2	0.06	10	RH	DRM	Y
Pergolizzi and Chua, 2016	A	On/Off	LPC	35	2	0.06	20	RH	MS	Y
Pisoni et al., 2015	A	On	LPPC	35	1.5	0.04	15	RH	S	Y
	A	On	LTC	35	1.5	0.04	15	RH	S	Y
Teo et al., 2011	A	Off	LdlPFC	35	1	0.03/0.06	20	CS	S	N
Teo et al., 2011	A	Off	LdlPFC	35	2	0.03/0.06	20	CS	S	N
Present study	A,S	On	LIFG	25	1.5	0.06	25	CS	-	-

A, anodal; ATL, anterior temporal lobe; C, cathodal; CS, contralateral supraorbital area; Do-Fr, dorso-frontal; DRM, Deese-Roediger-McDermott; Fpz, orbital midline; Fr-Te, frontal-temporal; IFG, inferior frontal gyrus; Iz, inion; L, left; M, mastoid; M1, primary motor cortex; MS, modified Sternberg; Off, offline; Off(+20), offline 20 min after stimulation cessation; On, online; PF, phonemic fluency; PPC, posterior parietal cortex; dlPFC, dorsolateral prefrontal cortex; T, temporal lobe; Te-Pa, temporal-parietal; LPC, parietal cortex; R, right; RC, right cerebellum; RD, right deltoid; RH, right homolog; S, Sternberg task; SF, semantic fluency; V, vertex. *15 s-on/15 s-off, with 2 s ramp up and 2 s ramp down.

#### Tasks

##### Verbal fluency

Parallel versions of semantic and phonemic fluency tasks were used in the two testing sessions. In each session, the experimenter presented a pseudo-random selection of two categories and two letters. For semantic fluency, semantic categories were: *Animals, Fruits, Super Market Items*, and *Musical Instruments*. For phonemic fluency, letters were *C, L, S*, and *A*. Our choice of semantic categories was based on previous tDCS experiments (see Cattaneo et al., 2011; Vannorsdall et al., 2012), whilst letters were chosen from the two most widely used phonemic fluency tasks (i.e., CLF or SAF and; for review, see Barry et al., 2008). Our decision was justified by the control participant data, which showed good correspondence across stimuli in each task (see Appendix 1 in Supplementary Material). Moreover, letters/categories were counterbalanced across testing session and stimulation condition across participants.

Participants were given 1 min to name as many unique words as possible that started with a given letter or belonged to a give semantic category. Proper names (e.g., *Rochester* or *Robert*) or repetitions (even with a different ending; e.g., *eat* followed by *eating*) were not allowed. Participants were reminded to keep going until the time ran out even if they drew a blank. To ensure participants understood the task, the experimenter provided an practice example (e.g., “*for the letter T I could say, “terrible,” “turn,” and “table”*) and asked participants if they could think of any other words. Responses were recorded using a voice recorder and scored after the testing session. Our primary outcome measure was the average number of words produced correctly, with repetitions and rule violations excluded. Slang words and foreign words were permissible answers so long as they were listed as Standard English words. Participants were asked to indicate the meaning of a word in instances of ambiguity (e.g., *frank*) at the end of the task (for a similar procedure, see Iyer et al., 2005; Cattaneo et al., 2011; Penolazzi et al., 2013a). We identified switches based on the protocol designed by Troyer et al. (1997; see also Troyer, 2000; Troyer and Moscovitch, 2006), which defines switches as the number of transitions between clusters, including single words. For full details on the protocol, see Appendix 3 in Supplementary Material.

##### Probe tasks

Participants were shown a list of words and then asked to make a yes/no decision about whether a test word, or *probe*, had appeared in the list. Responses were given by pressing keys *g* (for *yes*) or *j* (for *no*) using the index finger of the right hand. Participants were asked to give fast and accurate responses. List items were presented one after the other, each centered for 800 ms followed by a blank screen for 500 ms; probes were presented centered for 4,000 ms or until participants gave a response after which a blank screen followed for 1,500 ms before the next trial started. Words were presented in black Courier New typeface 18-font. Probes appeared in red ink to distinguish them from list items. Words were presented using E-Prime 2 Software and a Dell Laptop computer screen (screen size: 15.6″). Words were matched for word length and frequency (based on CELEX Database; Baayen et al., 1995; see Appendix 2 in Supplementary Material).

Lists were presented randomized across participants for the semantic-associated probe, but in a fixed order for the recent-probe because here probes must appear or not appear a number of lists back. In terms of scoring, for reaction time analysis, we excluded incorrect responses and reaction times below 250 ms or above 2.5 standard deviations from the participant mean.

***Recent-probe stimuli.*** We generated 5 word lists each composed of 8 words plus one probe word from a sample of 216 nouns repeated two or three times (for a total of 434 words if you include probes). There were two types of probes: *positive* (appeared in the word list; *n* = 21) and *negative* (did not appear in the list; *n* = 30). There were three types of negative probes, *negative* (which did not appear in the preceding two lists; *n* = 10), *recent-negative* (appeared in the immediately preceding list; *n* = 10), and *non-recent-negative* (appeared in the previous but one list; *n* = 10). To avoid floor effects, positive and negative probes were never presented in the eighth position in the list, and were distributed in all the other positions (positive probes = 3 per position; recent- and non-recent-negative probes = 1 or 2 per position). To generate parallel versions, stimuli were resampled to generate a new set of 51 word lists and paired probe items. Words were matched for word length and frequency (based on CELEX Database; Baayen et al., 1995; see Appendix 2 in Supplementary Material).

***Semantic probe stimuli***. We generated 180 word lists, each composed of five words plus one probe word from a pool of 982 nouns. Half were *positive* lists containing the probe (*n* = 90) and half were *negative* lists (*n* = 90) not containing the probe. Positive probes, were either *positive-related* (*n* = 40) or *positive-unrelated* (*n* = 50). For *positive-related*, the list included the probe plus one word semantically related to it (e.g., *plug, tunnel, wire, bishop, bracelet*; probe: *plug*); for *positive-unrelated*, the list included only the probe with no other related word (e.g., *bandage, shield, life, puff*, *worker*; probe: *bandage*). Negative lists also contained different types of probes. For ***negative-associated*** (*n* = 20), the list included two items semantically related to each other and to the probe (e.g., *valley, plum, violin, peach, shawl*: probe: *apricot*)*;* for ***negative-combined** (n* = 20), the list included two words that were unrelated to one another but whose meaning overlapped with the probe (e.g., *vehicle, lobe, lizard, jewel, hostage*: probe: *earring*), for ***negative associated-combined*** (*n* = 20), the list included two words which were related to one another *and* whose meaning overlapped with the probe (e.g., *cage*, ***book***, *law*, ***plot***, *plot*: probe: *novel*). For ***negative-unrelated*** (*n* = 30), the list did not include any items semantically related or whose meaning overlapped with the probe (e.g., *ball, table, wire, camel*: probe: *boat*). The order in which words were presented in each list was the same across participants. The selection of probe items and the list position of items related to the probe were controlled in the following manner to avoid floor effects. For positive-unrelated probes, 6 positive probes were taken from each position (i.e., 6 probes × 5 positions = 30 probes); for positive-related probes, 8 related items were positioned in each of the first 4 list positions (i.e., 8 probes × 4 positions = 30 probes); for negative-associated, negative-combined, negative associated-combined probes, the related items appeared always in the second position (*n* = 10) and then in the third and fourth position an equal number of times (*n* = 5). The first and last position were not probed to avoid primacy and recency effects. Parallel versions of the task were generated by equally dividing the lists between the two versions. Words in the two versions were matched for length across positions; probes were matched for both word length and frequency (based on CELEX Database; Baayen et al., 1995; see Appendix 2 in Supplementary Material).

##### Digit span

At the beginning of the first session, experimental participants completed the digit span task. They were read a sequence of single digits at a rate of one per second and asked to recite back the sequence in the order it was given, as soon as it was finished. Ten sequences of each length were presented. The task started with a sequence of *four*-digits and progressed up to *eight*-digit sequences. A participant moved on to longer sequences if at least 50% of the list for a given length were repeated correctly, otherwise testing was stopped. To compute the span, each sequence recalled correctly was scored as 0.1 (10 correct sequences for a given length = 1 point). Sequences up to four were assumed to be correct.

##### Motivational scale (BIS/BAS)

After the digit span, participants completed the BIS/BAS questionnaires (Carver and White, 1994). The Behavioral Inhibition System (BIS) scale measures responsiveness to aversive stimuli (e.g., “I have very few fears compared to my friends”). The Behavioral Approach System (BAS) scale measures responsiveness to reward and includes three sub-scales: (1) Reward responsiveness (*BAS-RR*; e.g., “It would excite me to win a contest”); (2) Drive (BAS-D; e.g., “I go out of my way to get things I want”); and, (3) Fun Seeking (FAS-FS; e.g., “I often act on the spur of the moment”; for further details on scale item, see Appendix 4 in Supplementary Material). Across the two scales, participants are asked to rate to what extent they agree or disagree with 24 statements using a four-point rating scale, ranging from 1 (“very true for me”) to 4 (“very false for me”). Participants were asked to respond to all items as accurately/honestly as possible, providing only one response to each item. It was stressed that each item should be considered on its own, to avoid participants making their responses “consistent.”

#### Participants

Sixty-three undergraduate students from Aston University participated for course credits or financial reimbursement, and were assigned to the experimental or control group in a semi-random fashion. One participant in the experimental group failed to turn up to the second stimulation session due to other commitments. This left 20 participants for recent probe (11 female; 20 ± 1.10); 19 participants for the semantic associated probe (9 female; 19 ± 1.00); and 24 participants for the control group (9 female; 21 ± 1.20). All participants were right-handed and native English speakers. We excluded volunteers with language impairments, history of migraine, headaches (frequent or severe), skin disorders (e.g., eczema), any adverse experience to previous tDCS, any history of epilepsy or stroke, head/metal implants, any neurological disorders, and any volunteers who had participated in a tDCS or TMS study in the 6 months prior to the current study.

#### Data re-sampling

##### Pseudo stimulation conditions from control data

For the experimental participants, we counterbalanced the order of stimulation (Sham vs. tDCS) and the task stimuli sets (A vs. B). Thus, in session one, half of participants received sham whilst the other half received active tDCS, and half of participants that received either form of stimulation saw stimuli set A whilst the other half saw B. In the control group—in which stimulation was not applied—half of participants saw set A or B in the first session (and vice versa in the second session). Thus, to make data from the control group comparable with data from the experimental group, we resampled data from the control group to create two pseudo datasets (referred to as, *pseudo-sham* and *pseudo-real*), with each dataset including data from the first and second testing session and from stimuli sets A and B (for the same method, see Westwood et al., 2017).

##### Division of participants into sub-groups

We generated subgroups based on the median baseline scores of working memory span (digit span scores), motivation (BAS-RR scores), switching (correct switches over total correct words generated), and interference control (differences between lure and non-lure trials). Switching and interference control were based on data recorded during sham stimulation (for similar method, see Hsu et al., 2016). For interference control, with the recent-probe task, we calculated the difference between negative probes and the average of recent-negative and non-recent-probes. With the semantic-associated probe task, we calculated the difference between negative unrelated probes and the average of negative-associated, combined, and associated plus combined trials.

#### Ethical approval

Our experimental investigation was approved by The Ministry of Defense Research Ethics Committee, and by the Aston Research Ethics Committee. All participants gave written informed consent prior to any testing session in accordance with the Declaration of Helsinki.

### Results for experiment 1: verbal fluency

#### Group analysis

##### Overall performance

**Figure 1:**
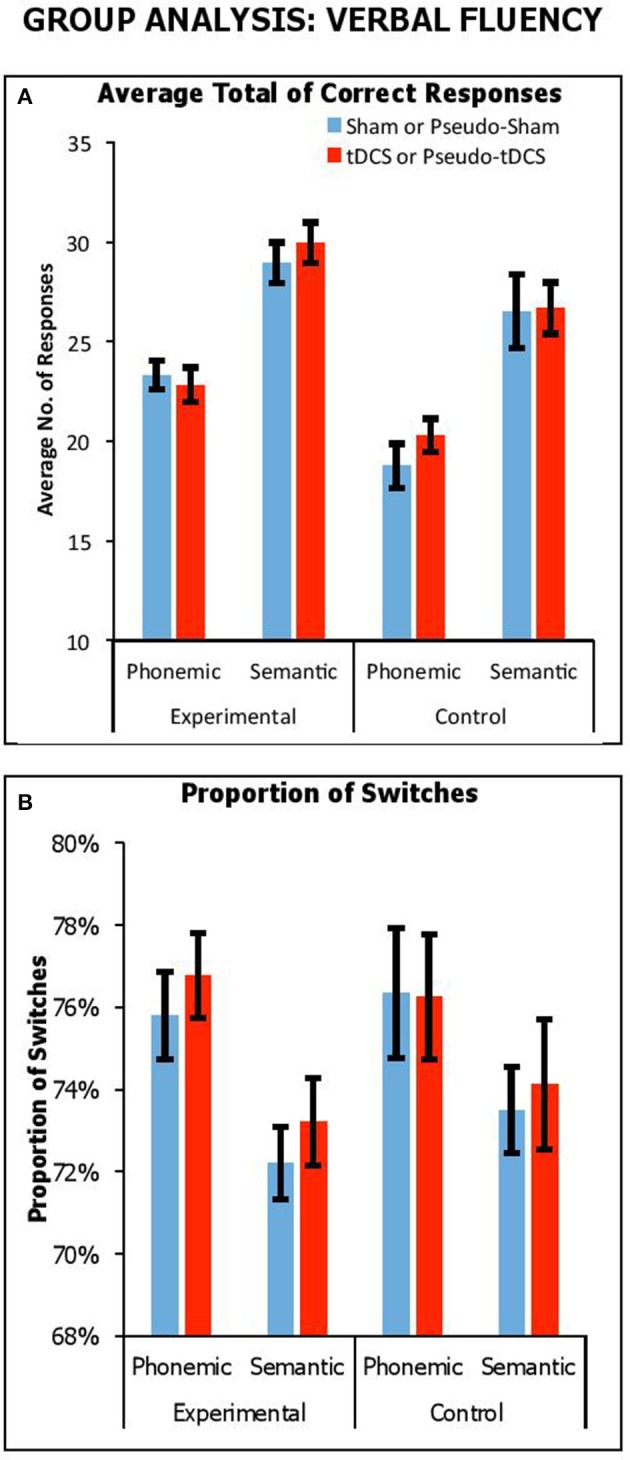
Performance across stimulation conditions (sham vs. tDCS conditions for experimental group; pseudo-sham vs. pseudo-tDCS for control group) and fluency tasks in terms of overall average no. of correct responses **(A)** and proportion of switches (i.e., average no. of switches over average response) expressed as a percentage **(B)**. Error Bars indicate Standard Error.

Figure 1A shows overall performance in terms of the average number of correct responses generated for semantic and phonemic fluency tasks across stimulation conditions and participant groups. We combined results from all participant groups to carry out a mixed factor ANOVA, with *Condition* (Real vs. Sham for experimental group; Pseudo-Real vs. Pseudo-Sham for control group) and *Fluency Task* (Phonemic vs. Semantic) as within-participants factors, and *Group* (Control vs. Experimental) as a between-participants factor. There was a significant main effect of *Fluency Task* [*F*_(1, 61)_ = 112, *p* < 0.001, η_*p*_^2^ = 0.65], with a greater number of responses generated in semantic fluency compared to phonetic fluency, as was expected (21.3 ± 0.5 vs. 28 ± 0.6, respectively). There was a significant main effect of *Group* [*F*_(1, 61)_ = 12.80, *p* = 0.001, η_*p*_^2^ = 0.17], with experimental participants providing more responses across both tasks than control participants (26.3 ± 0.6 vs. 23.1 ± 0.71, respectively). There was no interaction *Task* by *Group* [*F*_(1, 61)_ = 0.28, *p* = 0.60, η_*p*_^2^ = 0.01]. Importantly, there were no significant interactions with *Condition*, including *Condition* by *Group* [*F*_(1, 61)_ = 0.26, *p* = 0.62, η_*p*_^2^ = 0.004], *Condition* by *Task* [*F*_(1, 61)_ = 0.002, *p* = 0.97, η_*p*_^2^ = 0.02], and *Condition* by *Group* by *Task* [*F*_(1, 61)_ = 1.1, *p* = 0.30, η_*p*_^2^ = 0.02].

Thus, we observed that participants generally produced more responses in semantic fluency, which was expected since phonemic fluency is comparatively more effortful (for similar results, see Cattaneo et al., 2011; Vannorsdall et al., 2016), but importantly in no instance did tDCS improve performance relative to sham. We did find that the experimental group generated more responses compared to the control group, but this difference did not interact with stimulation condition. We assume this group difference arose from a placebo benefit in the response to the *presence*—not the modulatory effect—of tDCS.

##### Effect of stimulation on switching

Here, we considered the impact of tDCS on switching by looking at the proportion of correct switches relative to number of correct responses. Figure 1B shows proportions of switches (expressed as a percentage) across fluency tasks, stimulation conditions and participant groups. We combined results from all experiments and participant groups to carry out a mixed factor ANOVA, with *Condition* (Real vs. Sham for experimental group; Pseudo-Real vs. Pseudo-Sham for control group) and *Fluency Task* (Phonetic vs. Semantic) as within-participants factors, and *Group* (Control vs. Experimental) as a between-participants factor. We used proportion of switches as the dependent measure. There was a significant main effect of *Fluency Task* [*F*_(1, 61)_ = 21.42, *p* < 0.001, η_*p*_^2^ = 0.26], with a greater proportion of switches in phonemic fluency compared to semantic fluency (76.3 ± 0.6 vs. 73.3 ± 0.5%, respectively), as expected. There was no significant main effect of *Group* [*F*_(1, 61)_ = 0.35, *p* = 0.56, η_*p*_^2^ = 0.01], nor an interaction of *Condition* by *Group* [*F*_(1, 61)_ = 0.13, *p* = 0.72, η_*p*_^2^ = 0.002], of *Condition* by *Task* [*F*_(1, 61)_ = 0.02, *p* = 0.88, η_*p*_^2^ < 0.001], and of *Task* by *Group* [*F*_(1, 61)_ = 0.66, *p* = 0.42, η_*p*_^2^ = 0.01]. Crucially, there was no significant three-way interaction of *Condition* by *Group* by *Task* [*F*_(1, 61)_ = 0.02, *p* = 0.88, η_*p*_^2^ < 0.001]. Thus, although the demands on switching were greater in phonemic than semantic fluency, which was expected given the difficulty to produce a cluster in this task, switching ability was not modulated by tDCS.

#### Subgroup analysis

##### Effect of working memory span, reward sensitivity and switching ability

**Figure 2:**
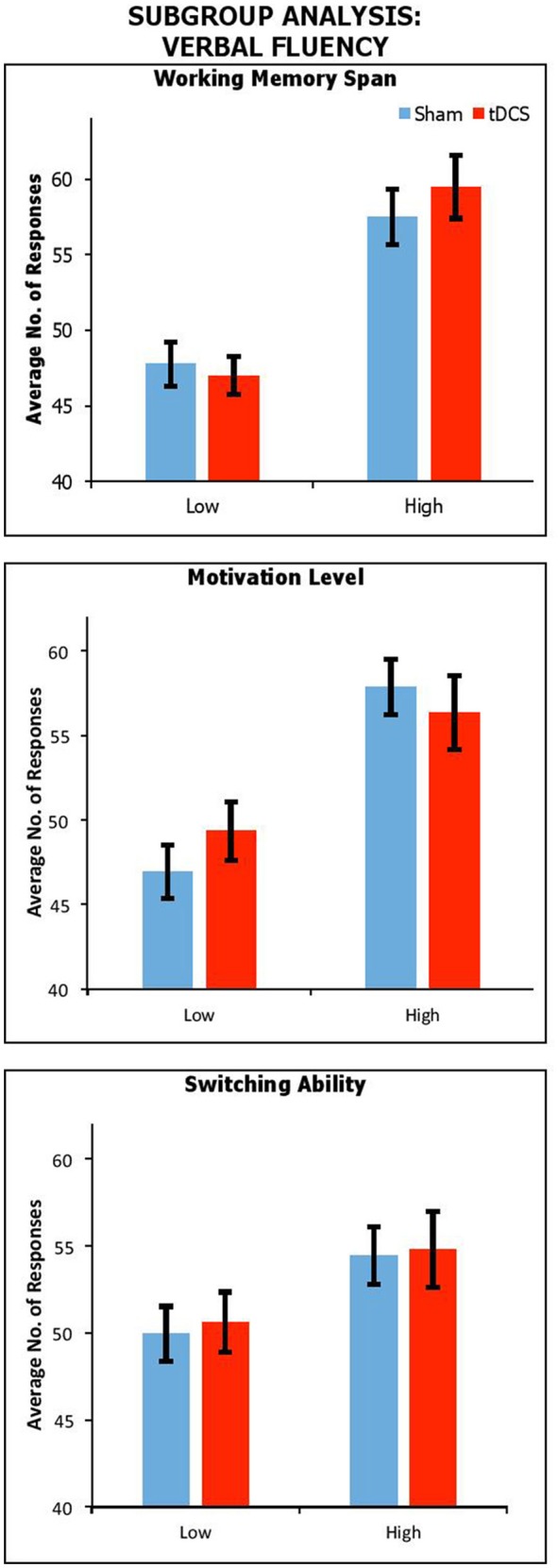
Performance across stimulation conditions, with participants subdivided by Working Memory Span; Motivation Level; and Switching Ability. Error Bars indicate Standard Error.

Figure 2 shows performance under sham and anodal tDCS with respect to variation in working memory span, motivation, and switching ability. We carried out a series of mixed factors ANOVAs, which included *Condition* (Real vs. Sham) as within-participants factors, *Span, BAS-R* or *Switching* (High vs. Low) as a between-participants factor. We do not include control data because here only the experimental group is of interest. The results showed a significant main effect of *Span* [*F*_(1, 37)_ = 35.91, *p* < 0.001, η_*p*_^2^ = 0.49], *BAS-R* [*F*_(1, 37)_ = 16.25, *p* < 0.001, η_*p*_^2^ = 0.31], and a trendwise—but not significant—main effect of *Switching* [*F*_(1, 37)_ = 3.02, *p* = 0.09, η_*p*_^2^ = 0.08]. There was no significant interactions of *Condition* by *Span* [*F*_(1, 37)_ = 1.57, *p* = 0.22, η_*p*_^2^ = 0.04], by *BAS-R* [*F*_(1, 37)_ = 1.02, *p* = 0.32, η_*p*_^2^ = 0.03], or by *Switching* [*F*_(1, 37)_ = 0.14, *p* = 0.96, η_*p*_^2^ < 0.001].

### Results for experiment 2: probe tasks

#### Group analysis

##### Overall performance

**Figure 3:**
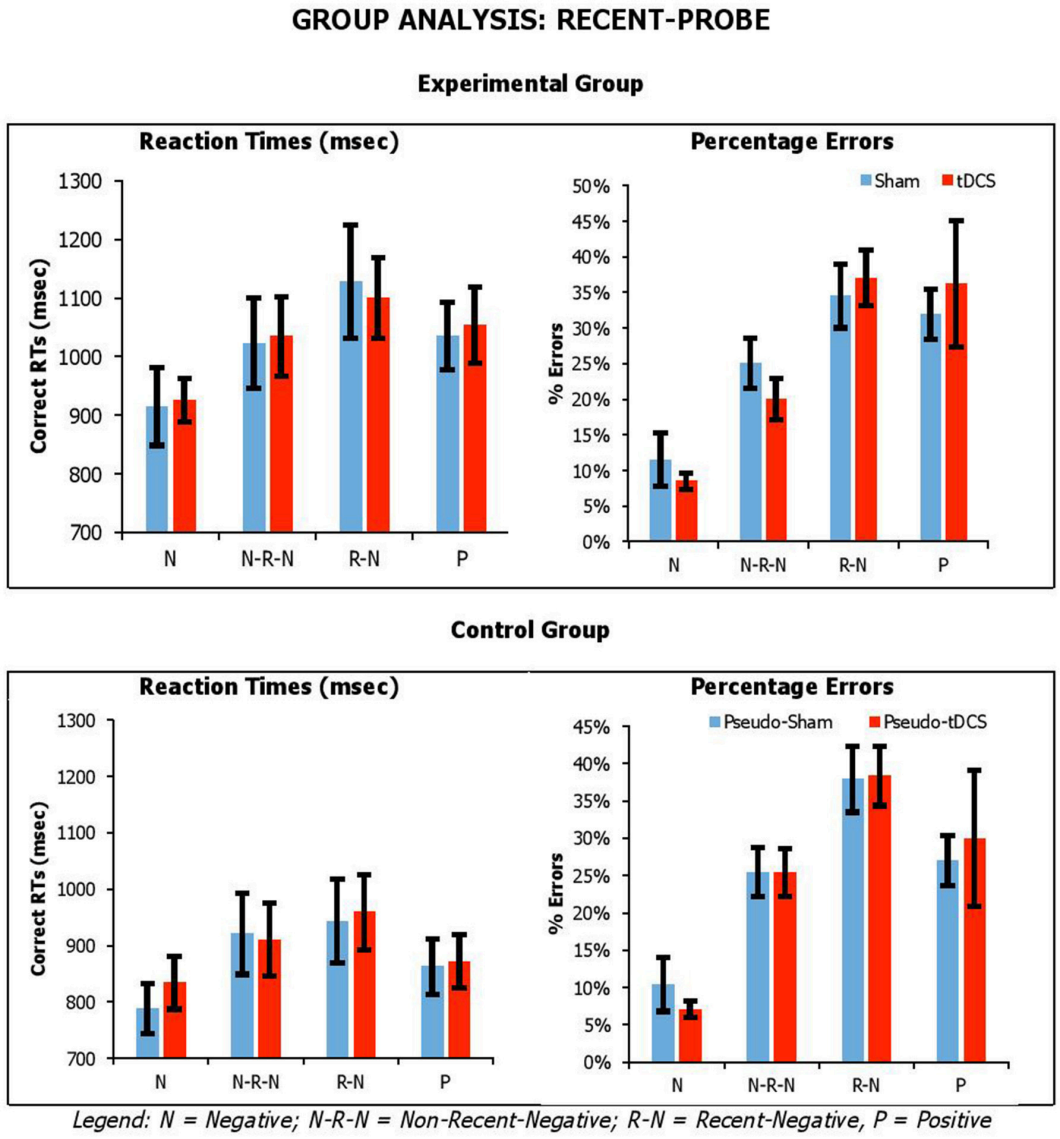
Average correct RTs (ms) and percentages errors for recent-probe task across participant groups, probe conditions, and stimulation conditions. Error Bars indicate Standard Error.

**Figure 4:**
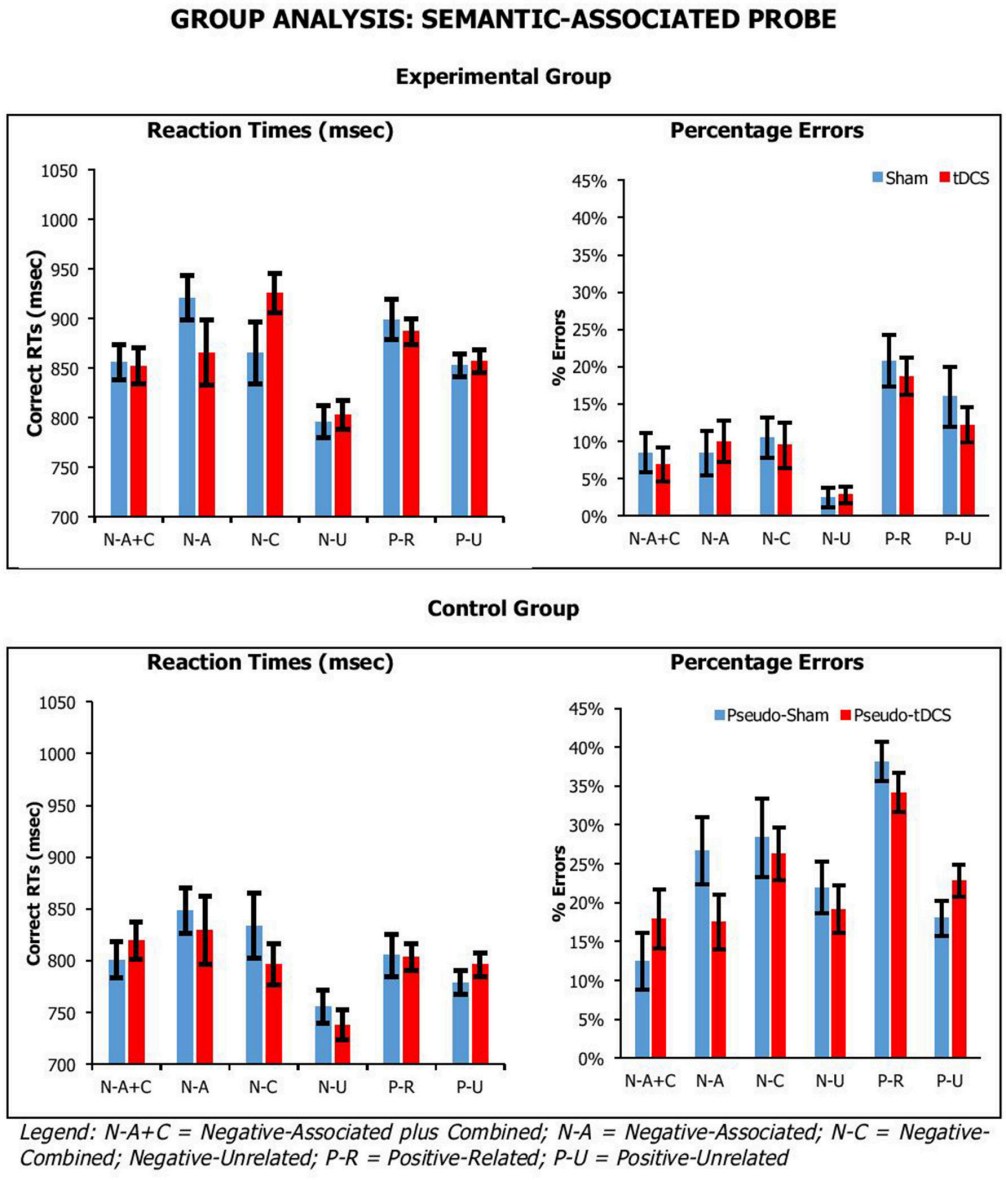
Average correct RTs (ms) and percentages errors for semantic-associated probe across participant groups, probe conditions, and stimulation conditions. Error Bars indicate Standard Error.

Figures 3, 4 show the average performance across participant groups for each probe task, probe type and stimulation condition. For each probe task, we carried out mixed factor ANOVAs combing data from both participant groups, with *Condition* (Sham vs. tDCS for experimental group; Pseudo-Sham vs. Pseudo-tDCS for control group) and *Probe Conditions* (for *recent-probe*, negative, non-recent-negative, recent-negative, positive; for *semantic-associated probe*, negative-associated, negative-combined, negative associated-combined, negative-unrelated, positive-unrelated, positive-related) as within-participants factors, and *Group* (Control vs. Experimental) as a between-participants factor. Separate analyses were carried out on RTs and errors.

For the *recent-probe*, we found a significant main effect of *Probe Condition* [_RTs_*F*_(3, 126)_ = 17.83, *p* < 0.001, η_*p*_^2^ = 0.30; _errors_*F*_(3, 126)_ = 44.70, *p* < 0.001, η_*p*_^2^ = 0.52], with positive probe being overall responded to faster but less accurately than negative probes; and *Group* for reaction times but not errors [_RTs_*F*_(1, 42)_ = 4.09, *p* = 0.05, η_*p*_^2^ = 0.09; _errors_*F*_(1, 42)_ = 0.003, *p* = 0.96, η_*p*_^2^ < 0.001], with the experimental group being slower than the control group (1,032 ± 53 vs. 886 ± 49). The *Probe Condition* by *Group* interaction was significant for errors, but not reaction times [_RTs_*F*_(3, 126)_ = 1.08, *p* = 0.36, η_*p*_^2^ = 0.03; _errors_*F*_(3, 126)_ = 9.91, *p* < 0.001, η_*p*_^2^ = 0.19]. Crucially, there was no significant interaction of *Condition* by *Group* [_RTs_*F*_(1, 42)_ = 0.02, *p* = 0.89, η_*p*_^2^ < 0.001; _errors_*F*_(1, 42)_ = 0.002, *p* = 0.96, η_*p*_^2^ < 0.001], *Condition* by *Probe Condition* [_RTs_*F*_(3, 126)_ = 0.31, *p* = 0.82, η_*p*_^2^ = 0.01; _errors_*F*_(3, 126)_ = 1.24, *p* = 0.30, η_*p*_^2^ = 0.03], or *Condition* by *Group* by *Probe Condition* [_RTs_*F*_(3, 126)_ = 0.34, *p* = 0.80, η_*p*_^2^ = 0.01; _errors_*F*_(3, 126)_ = 0.38, *p* = 0.77, η_*p*_^2^ = 0.01].

For the *semantic-associated probe*, we found a significant main effect of *Probe Condition* [_RTs_*F*_(5, 205)_ = 5.93, *p* < 0.001, η_*p*_^2^ = 0.13; _errors_*F*_(5, 205)_ = 24.63, *p* < 0.001, η_*p*_^2^ = 0.38], with positive probe being overall responded to faster but less accurately than negative probes; and *Group* [_RTs_*F*_(1, 41)_ = 4.41, *p* = 0.04, η_*p*_^2^ = 0.10; _errors_*F*_(1, 41)_ = 39.18, *p* < 0.001, η_*p*_^2^ = 0.49], with control participants being significantly faster and more error prone than experimental participants (869 ± 25 vs. 798 ± 23 ms; 11 ± 2 vs. 24 ± 1%, respectively). There was a significant interaction for *Probe Condition* by *Group* for percentage errors, but not reaction times [_RTs_*F*_(5, 205)_ = 0.35, *p* = 0.89, η_*p*_^2^ = 0.03; _errors_*F*_(5, 205)_ = 3.13, *p* = 0.01, η_*p*_^2^ = 0.07]. Crucially, there were no significant interactions of *Condition* by *Group* [_RTs_*F*_(1, 41)_ = 0.09, *p* = 0.77, η_*p*_^2^ = 0.002; _errors_*F*_(1, 41)_ = 0.01, *p* = 0.94, η_*p*_^2^ = 0.00], *Condition* by *Probe Condition* [_RTs_*F*_(5, 205)_ = 1.08, *p* = 0.37, η_*p*_^2^ = 0.03; _errors_*F*_(5, 205)_ = 0.71, *p* = 0.62, η_*p*_^2^ = 0.02], or *Condition* by *Group* by *Probe Condition* [_RTs_*F*_(5, 205)_ = 1.33, *p* = 0.26, η_*p*_^2^ = 0.03; _errors_*F*_(5, 205)_ = 2.12, *p* = 0.06, η_*p*_^2^ = 0.05].

Overall, our tasks were sensitive to our manipulation, which we assume is related to executive selection. Relative to negative (neutral) probe trials, longer reaction times and higher percentage errors were observed on lures. This was confirmed by planned paired-samples *t*-tests that considered separately data from the control group and experimental group. In recent-probe, relative to negative probes, there was a significant difference with non-recent-probes, recent-probes and positive probes (values across groups: RTs, *t* = −2.85–5.10, *p* < 0.009; errors, *t* = −5.29–9.84, *p* < 0.001). In semantic probe, relative to negative-unrelated probes, there was a significant difference negative-associated, negative-combined, negative-combined-plus-associated, positive-unrelated, and positive-related (values across groups: RTs, *t* = −2.76–5.25, *p* < 0.01; errors, *t* = −1.09–7.05, *p* < 0.02), although for errors in the control group there were no significant differences for negative-associated, negative-combined-plus-associated, and positive-unrelated. A similar pattern was seen with positive probes. Importantly, tDCS did not systematically modify performance.

##### Effect of stimulation on aggregated interference

**Figure 5:**
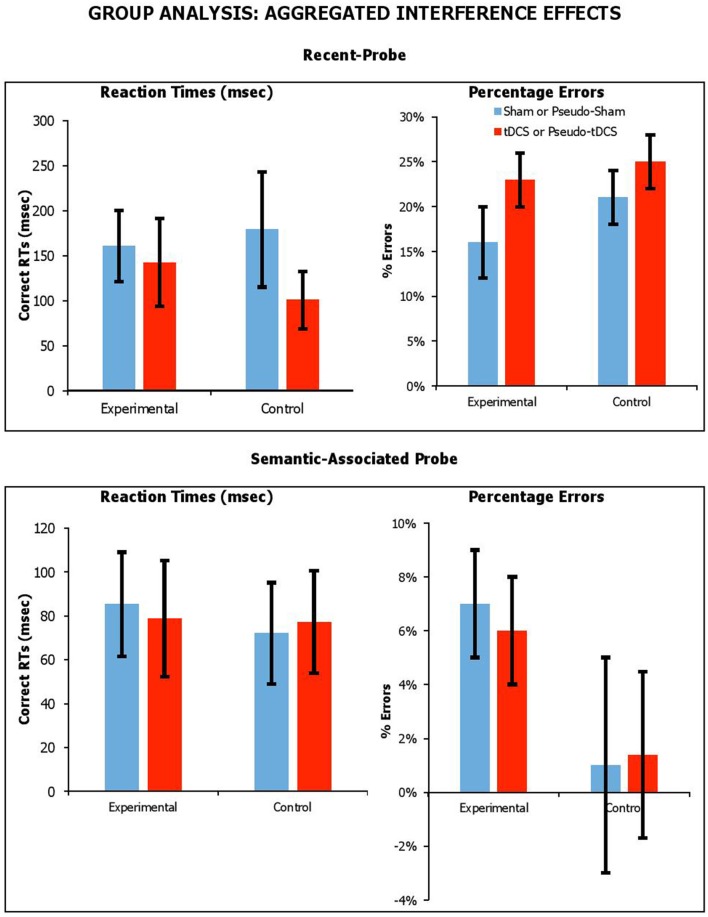
Aggregated interference across probe tasks (recent-probe, upper panel; semantic-associated probe, lower panel) stimulation conditions and participant groups. Interference measured as difference between negative probes and the average of recent-negative and non-recent-probes or as difference between negative unrelated probes with the average of negative-associated and -combined. Error Bars indicate Standard Error.

We thought the effects of tDCS might be detectable if the interference effect from lures were aggregated across lure conditions. For recent-probe, we calculated aggregated interference as the difference between neutral negative probes and the average of recent-negative and non-recent-negative probes. For the semantic-associated probe, we calculated aggregated interference as the difference between neutral/unrelated negative probes with the average of negative-associated, negative-combined and negative-associated-combined probes. The aggregated interference effects across probe tasks, participant groups and stimulation conditions are presented in Figure 5 for RTs and errors.

We carried out separate mixed factor ANOVAs, with *Condition* as a within-participants factor, *Group* (Experimental vs. Control) as a between-participants factor, and *aggregated interference* as a dependent measure for each task and separately for RTs and errors. With the *recent-probe*, there was no main effect of *Group* [_RTs_*F*_(1, 42)_ = 0.05, *p* = 0.83, η_*p*_^2^ = 0.01; _errors_*F*_(1, 42)_ = 2.35, *p* = 0.13, η_*p*_^2^ = 0.05], and no significant *Condition* by *Group* interaction [_RTs_*F*_(1, 42)_ = 0.50, *p* = 0.48, η_*p*_^2^ = 0.01; _errors_*F*_(1, 42)_ = 0.01, *p* = 0.92, η_*p*_^2^ < 0.001]. With the *semantic-associated probe*, there was no main effect of *Group* [_RTs_*F*_(1, 41)_ = 0.12, *p* = 0.73, η_*p*_^2^ = 0.003; _errors_*F*_(1, 41)_ = 2.87, *p* = 0.10, η_*p*_^2^ = 0.07], and no significant *Condition* by *Group* interaction [_RTs_*F*_(1, 41)_ = 0.05, *p* = 0.83, η_*p*_^2^ = 0.001; _errors_*F*_(1, 41)_ = 0.02, *p* = 0.90, η_*p*_^2^ = 0.00]. Thus, it was clear that tDCS had no systematic effect on the magnitude of aggregated interference.

#### Subgroup analysis

##### Effect of working memory span, reward sensitivity and interference control

**Figure 6:**
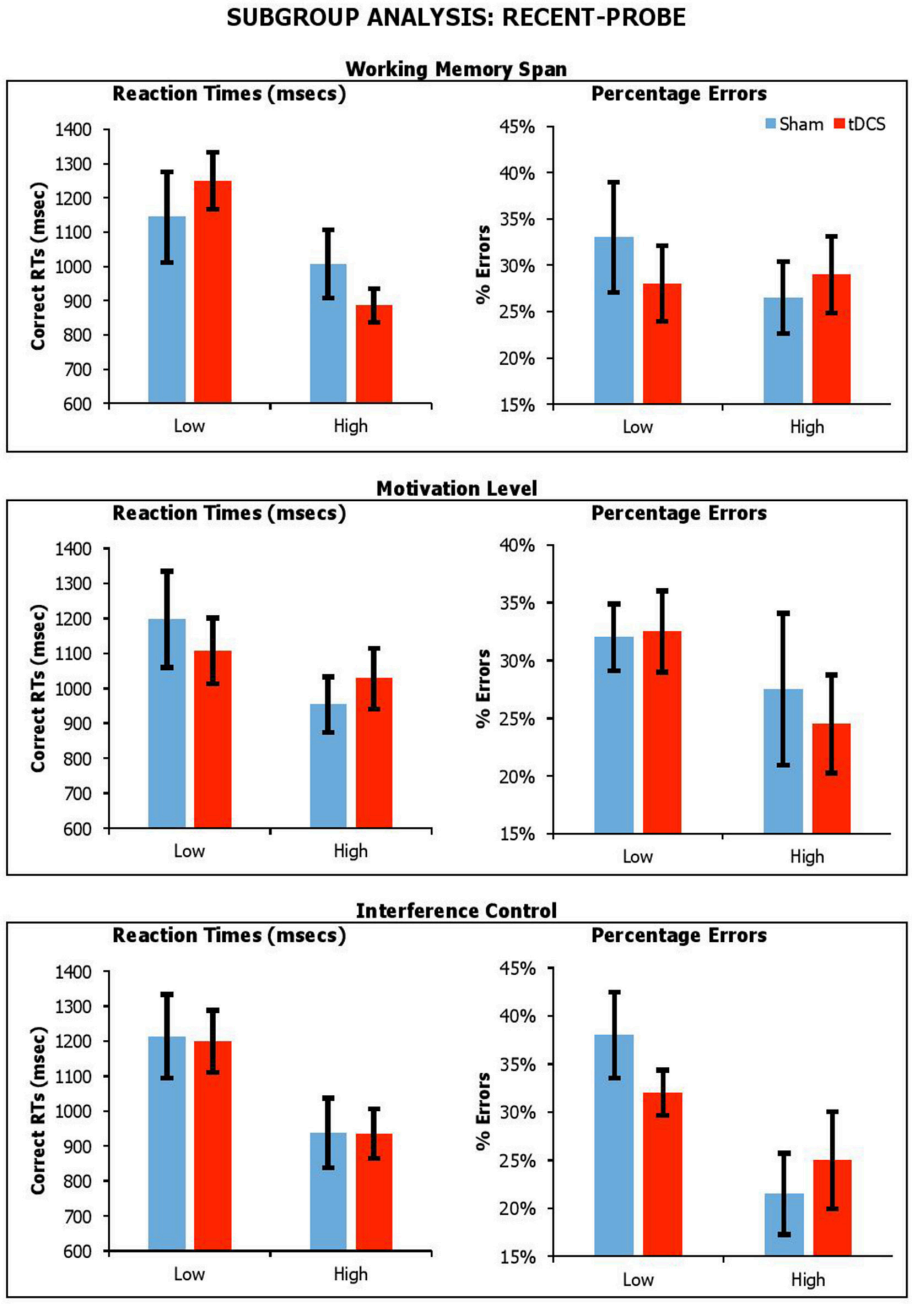
Performance on lure trials (i.e., average of non-recent-negative and recent-negative) in recent-probe across stimulation conditions, with participants divided by Working Memory Span, Motivation Level, and Interference Control. Error Bars indicate Standard Error.

**Figure 7:**
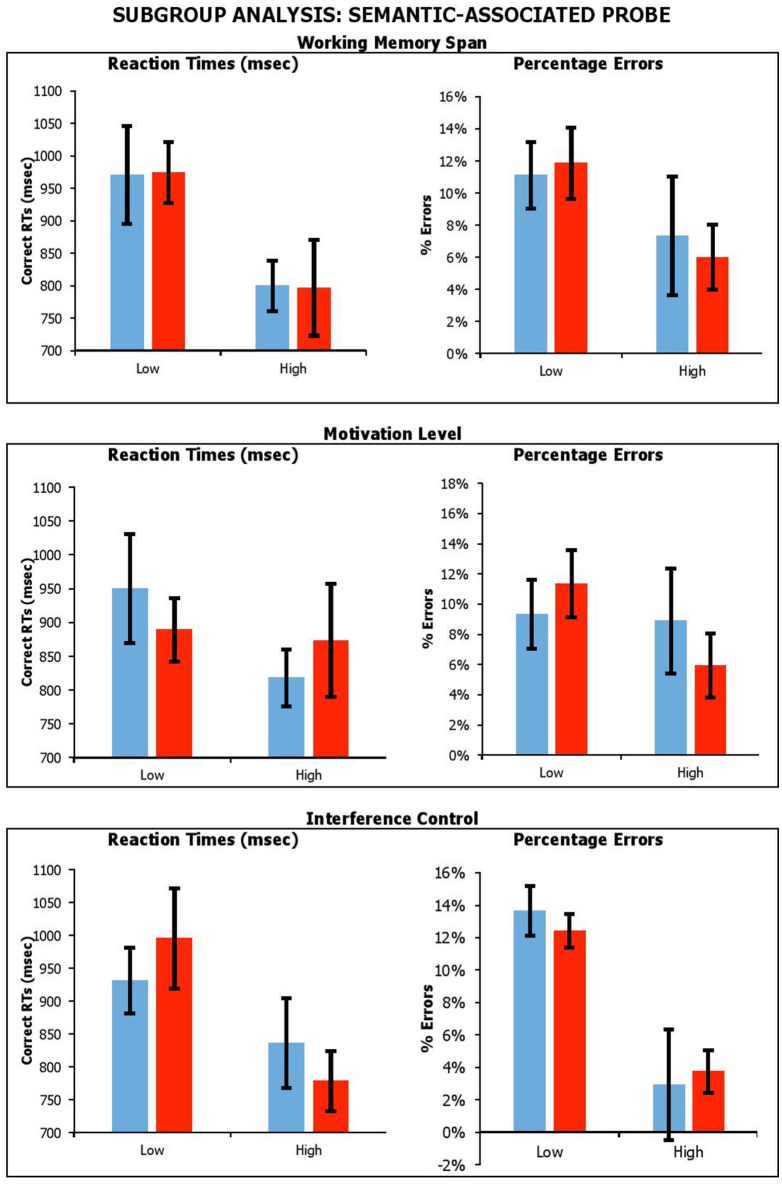
Performance on lure trials (i.e., average of negative-associated, negative-combined and negative associated-combined) in semantic-associated probe across stimulation conditions, with participants divided by Working Memory Span; Motivation Level; and Interference Control. Error Bars indicate Standard Error.

Figures 6, 7 shows performance under sham and anodal tDCS with respect to variation in working memory span, motivation, and switching ability. We carried out a series of mixed factors ANOVAs, which included *Condition* (Real vs. Sham) as within-participants factors, *Span, BAS-R* or *Interference Control* (High vs. Low) as a between-subjects factor, and *average performance across lure probes* as the dependent measure. Average performance across lure trials was calculated as follows. For recent probe, we averaged data across non-recent-negative and recent-negative probes. For semantic-associated probe, we averaged data across negative-associated, -combined, and -associated-combined. We do not include control data here because the experimental group is of interest.

With the *recent-probe*, the results showed significant main effects *Span* for reaction times only [_RTs_*F*_(1, 18)_ = 5.63, *p* = 0.03, η_*p*_^2^ = 0.24; _errors_*F*_(1, 18)_ = 0.22, *p* = 0.64, η_*p*_^2^ = 0.01], with participants with higher span being faster. There was a main effect of *Interference Control*, with participants with high control abilities being faster and more accurate [_RTs_*F*_(1, 18)_ = 6.92, *p* = 0.02, η_*p*_^2^ = 0.23; _errors_*F*_(1, 18)_ = 5.18, *p* = 0.04, η_*p*_^2^ = 0.22]. There were no main effect of *BAS-R* [_RTs_*F*_(1, 18)_ = 1.98, *p* = 0.18, η_*p*_^2^ = 0.10; _errors_*F*_(1, 18)_ = 1.21, *p* = 0.29, η_*p*_^2^ = 0.06]. There were no significant interactions of *Condition* by *Span* [_RTs_*F*_(1, 18)_ = 1.76, *p* = 0.20, η_*p*_^2^ = 0.09; _errors_*F*_(1, 18)_ = 1.68, *p* = 0.21, η_*p*_^2^ = 0.09], *Condition* by *BAS-R* [_RTs_*F*_(1, 18)_ = 0.88, *p* = 0.36, η_*p*_^2^ = 0.05; _errors_*F*_(1, 18)_ = 0.34, *p* = 0.57, η_*p*_^2^ = 0.02], and *Condition* by *Interference Control* [_RTs_*F*_(1, 18)_ = 0.004, *p* = 0.95, η_*p*_^2^ < 0.001; _errors_*F*_(1, 18)_ = 0.17, *p* = 0.69, η_*p*_^2^ = 0.01].

With the semantic-associated probe, the results showed a main effect of *Span* for reaction times only, with individuals with a higher span being faster [_RTs_*F*_(1, 17)_ = 6.99, *p* = 0.02, η_*p*_^2^ = 0.29; _errors_*F*_(1, 17)_ = 2.61, *p* = 0.13, η_*p*_^2^ = 0.13]. There was a significant main effect of *Interference Control*, with participants with higher control producing fewer errors, but there was no equivalent benefit on reaction times [_RTs_*F*_(1, 17)_ = 2.92, *p* = 0.11, η_*p*_^2^ = 0.15; _errors_*F*_(1, 17)_ = 18.65, *p* < 0.001, η_*p*_^2^ = 0.52], with individual with higher interference control being slower. There was no main effect of *BAS-R* [_RTs_*F*_(1, 17)_ = 0.28, *p* = 0.61, η_*p*_^2^ = 0.02; _errors_*F*_(1, 17)_ = 0.84, *p* = 0.37, η_*p*_^2^ = 0.05]. Importantly, there were no significant interactions of *Condition* by *Span* [_RTs_*F*_(1, 17)_ = 0.003, *p* = 0.96, η_*p*_^2^ < 0.001; _errors_*F*_(1, 17)_ = 0.25, *p* = 0.63, η_*p*_^2^ = 0.01], *Condition* by *BAS-R* [_RTs_*F*_(1, 17)_ = 0.79, *p* = 0.39, η_*p*_^2^ = 0.05; _errors_*F*_(1, 18)_ = 1.91, *p* = 0.19, η_*p*_^2^ = 0.10], or *Condition* by *Interference Control* interaction [_RTs_*F*_(1, 17)_ = 1.65, *p* = 0.22, η_*p*_^2^ = 0.09; _errors_*F*_(1, 17)_ = 0.29, *p* = 0.60, η_*p*_^2^ = 0.02].

#### Integrity of blinding

Stimulation was tolerated well by participants, with no adverse effects or withdrawals from the study because of stimulation. The feedback questionnaire showed that participants reported sensations commonly reported in other studies, which included tingling, itching, prickling, and burning/heat. All were rated as being of mild to moderate intensity, and neither the number of reported sensations or their intensity significantly differed between tDCS and sham conditions. Within-participant samples *t*-tests showed that participants guessed what stimulation they had been administered at chance level (χ^2^ = 0.29, *df* = 1, *p* = 0.59).

## Discussion

Recent reports of inconsistent outcomes in studies using single sessions of tDCS in healthy participants (e.g., Horvath et al., 2015; Westwood and Romani, 2017) may reflect the confounding impact of individual variation in cortical activity and/or level of ability on the response to tDCS. Thus, we carried out a series of experiments to see whether tDCS could modify performance on tasks that probe executive selection abilities, namely verbal fluency and probe tasks, considering the potential effects of individual variables. We used conventional stimulation parameters—i.e., 1.5mA anodal tDCS applied to the LIFG for 25 min during task performance. We expected that performance would be enhanced, given that these tasks involve executive control and that the LIFG is widely regarded to mediate this function. In particular, several lines of research show the LIFG supports verbal fluency and probe task performance (Hirshorn and Thompson-Schill, 2006; Badre and Wagner, 2007; Badre, 2008; Öztekin et al., 2009; Robinson et al., 2012; Biesbroek et al., 2016). We further explored whether the tDCS effect could be mediated by individual variation in overall performance in our tasks, digit span and more direct measures of executive function and motivational levels. Despite our efforts, we found no systematic effect of stimulation.

Our results are consistent with reports that highlight the problem of inconsistent outcomes in studies attempting to modulate performance in healthy participants with single sessions of anodal tDCS. Differences in outcomes may reflect the variation in stimulation protocols across studies, but there does not seem to be a consistent association between a particular protocol and positive results. Table 1 shows a comparison between the protocols used in our Experiments 1 and 2 and similar published studies. The protocol adopted by Marshall et al. (2005) is perhaps the most salient exception to the most common protocol, targeting the left/right dlPFC simultaneously with intermittent tDCS. Our study differs in the site and timing of stimulation, but our decision to target the LIFG using online stimulation should have increased, not decreased, the effectiveness of tDCS. Neuroimaging studies report greater cortical excitation during rather than after tDCS administration (Rae et al., 2013; Stagg et al., 2013; Martin et al., 2014), and online effects are thought to operate on neuronal populations activated by the task (Bikson and Rahman, 2013; Lapenta et al., 2013; Pisoni et al., 2017). In other key aspects (e.g., current density, site of reference electrode, stimulation duration), our parameters are well within the range used by previous studies.

Differently from us, other studies targeted the left temporal lobe. Three studies have reported positive effects with left temporal anodal tDCS, with a significant reduction in false memories in the DRM task (Boggio et al., 2009; Díez et al., 2017) and better performance in a recent-probe without lures (Pisoni et al., 2015). The left temporal lobe is important in lexical access, which is required for our tasks. However, for fluency and probe tasks top-down frontal selection mechanism are also likely to be engaged since in these tasks there is a special need to move from one lexical/semantic field to another (fluency tasks) and/or to inhibit distractors (probe tasks). In fact, neuroimaging studies show that frontal regions work in concert with temporal regions to mediate performance in these tasks (e.g., Badre, 2008; Biesbroek et al., 2016). One may assume, therefore, that temporal stimulation would improve performance on verbal fluency and item recognition tasks, by facilitating lexical retrieval and/or maintenance of task relevant information. Boosting selection control mechanisms, however, should also have a positive effect.

Clearly, the null effects we report may still be a result of the failure to use *optimal combination* of parameters, but the fact is that conditions in which reliable effects of tDCS can be measured have not been established, at least within conditions covered by our study (e.g., fluency, working memory, healthy participants, and one session of anodal tDCS). A good way to test the possibility that null effects were due to protocol differences—and to elucidate conditions in which tDCS can operate optimally—is to conduct direct replications of studies that report positive effects. In yet unpublished work, we failed to replicate Cattaneo et al. (2011), which reported a large positive effect on semantic and phonemic fluency tasks after anodal tDCS was applied to the LIFG. We encourage others to confirm the reliability of previous findings by way of direct or conceptual replication.

Lack of power could be another reason for null outcomes because it reduces the likelihood of finding a true effect, if one exists. The sample sizes used in our studies (*n* = 19 and 20) are relatively small, but consistent with previous studies that found positive effects on fluency and working memory (see Price et al., 2015; Mancuso et al., 2016). Our aggregated sample size from Experiment 1 (*n* = 39), gave us good power to detect a large (0.8) and medium (0.5) effect size (1-β = 0.99 and 0.86, respectively), but we had limited power to detect a small effect size (0.2; 1-β = 0.22, α = 0.05). A meta-analysis is an ideal means to evaluate effects across individual studies that are underpowered. Mancuso et al. (2016) reported results indicating that effects on working memory tasks are generally small or non-significant even with a large sample of 471 (Hedges' *g* = ~0.2, see left dlPFC analysis). Price et al. (2015), however, reported a significant mean effect size of roughly 0.5 (Hedges' *g*) with a large sample (*n* = 119) across studies measuring verbal fluency and word learning tasks. In yet unpublished work, we pooled data from Price et al., and several studies published since, including our data from Experiment 1. The results showed that with a sample of roughly 230 participants, anodal tDCS significantly improved fluency performance. Still, this effect was more moderate than reported by Price et al. (2015; roughly 0.3, Hedges' *g*), and potentially inflated by exceptionally large treatment effects from underpowered studies. Thus, it remains to be seen if, for fluency tasks, tDCS effects are stable for properly powered studies.

Finally, we used the BIS/BAS motivational scale to assess whether the effect of tDCS may interact with reward sensitivity. One possible alternative approach would be to use a measure that is directly related to the task. The goal of the paper, however, was to identify general moderators that may serve to refine conventional protocols, and trait reward sensitivity is an ideal candidate because high BAS scores are associated with performance on working memory and cognitive control functions (for reviews, see Gray and Burgess, 2004; Jonides and Nee, 2006; Savine et al., 2010; Fino et al., 2014). More importantly, however, we chose the BAS scale because it has previously been used to identify responders to tDCS modulation (see Metuki et al., 2012; Sela et al., 2012). Another possibility is that we manipulate the extent to which a task is rewarding. Only one study—to our knowledge—has investigated this, and found that a financial incentive improved the effect of anodal tDCS (*p* = 0.04, see Jones et al., 2015). It might be that an external motivation to do well on a task can boost the facilitatory effect of tDCS.

## Conclusions

We focused on single sessions of tDCS in healthy individuals and found negative results. We do not want, however, to dismiss possible stronger effects of tDCS in other conditions. Cortical excitability in healthy brains is potentially already at optimal levels, meaning that null effects may be due to ceiling effects. More reliable effects may be seen when anodal stimulation is compared with cathodal stimulation which should decrease performance (for review, see Jacobson et al., 2012; Horvath et al., 2015). It remains likely, however, that, in healthy brains, homeostatic mechanisms may reduce or even nullify the effect of tDCS in order to maintain stable network activity (Krause and Kadosh, 2014). Positive effects may be more likely in participants with pathological or reduced levels of excitability. For example, more consistent effects of tDCS have been reported in patients with aphasia (see Monti et al., 2013; de Aguiar et al., 2015; Elsner et al., 2015; Sandars et al., 2015; Shah-Basak et al., 2015; Cappon et al., 2016; Crinion, 2016). Stronger effects may also occur in tasks where processes and representations are not yet stable, such as in the case of learning. Lastly, positive effects may be more likely when tDCS is applied across repeated sessions, thereby allowing for effects to accumulate (Alonzo et al., 2012; Meinzer et al., 2014). Indeed, a number of studies have shown enhanced learning following repeated stimulation even in normal participants (Flöel et al., 2008; Dockery et al., 2009; Reis et al., 2009; Kadosh et al., 2010; Meinzer et al., 2014). Our study should encourage further studies to establish the conditions where tDCS effects are stronger and/or more reliable.

## Author contributions

SW and CR: designed the experiments; SW: carried out data collection and analysis; SW and CR: wrote the manuscript.

### Conflict of interest statement

The authors declare that the research was conducted in the absence of any commercial or financial relationships that could be construed as a potential conflict of interest.
