# Supplementary material for: Null Effects on Working Memory and Verbal Fluency Tasks When Applying Anodal tDCS to the Inferior Frontal Gyrus of Healthy Participants
Source: Front Neurosci. 2018 Mar 19;12:166. doi: 10.3389/fnins.2018.00166 (PMC5867342; doi:10.3389/fnins.2018.00166)
Supplement: Supplementary file 1 [file DataSheet1.docx]

## APPENDICES

### Appendix 1

| Table 1: table showing average correct responses for each letter/category used in fluency tasks, based on control data. Mean (Standard Deviation). | | | |
| --- | --- | --- | --- |
| Phonemic Fluency | | | |
| A | C | L | S |
| 8.9 (2.7) | 10.8 (1.4) | 8.9 (2.0) | 10.5 (2.7) |
|  |  |  |  |
| Semantic Fluency | | | |
| Animals | Fruits | Musical Instruments | Super Market |
| 16.3 (2.6) | 12 (2.6) | 13.1 (2.8) | 14.8 (2.4) |

### Appendix 2 – Lexical variables (word length and frequency, CELEX Database, Baayen et al., 1995) used in all versions of the recent-probe (Table 1) and semantic probe task (Table 2).

| Table 1: lexical variables for recent-negative probe (Version A and B); Mean (Standard Deviation). Version A was used in Experiment 1. | | | | |
| --- | --- | --- | --- | --- |
| Version A | N | R-N | N-R-N | P |
| List items |  |  |  |  |
| Length | 4 (1) | 4 (1) | 4 (1) | 4 (1) |
| Probes |  |  |  |  |
| Frequency | 191 (335) | 282 (766) | 122 (217) | 91 (144) |
| Length | 4 (1) | 4 (1) | 4 (1) | 4 (1) |
| Version B |  |  |  |  |
| List items |  |  |  |  |
| Length | 4 (1) | 4 (1) | 4 (1) | 4 (1) |
| Probes |  |  |  |  |
| Frequency | 72 (84) | 80 (130) | 66 (82) | 50 (60) |
| Length | 4 (1) | 4 (1) | 4 (1) | 4 (1) |
| Legend: N = negative; R-N = recent-negative; N-R-N = non-recent-negative; P = positive | | | | |

| Table 2: lexical variables for semantic-associated probe (Version A and B); Mean (Standard Deviation) | | | | | | |
| --- | --- | --- | --- | --- | --- | --- |
| Version A | N-A/C | N-A | N-C | N-U | P-R | P-U |
| List items |  |  |  |  |  |  |
| Length | 5.4 (.5) | 5.8 (.3) | 5.7 (.6) | 5.5 (.2) | 5.2 (.3) | 5.7 (.2) |
| Probe |  |  |  |  |  |  |
| Frequency | 59 (112) | 55 (112) | 24 (20) | 25 (26) | 47 (47) | 67 (124) |
| Length | 5.7 (1.01) | 5.5 (2) | 5.6 (1) | 5.3 (1) | 5.1 (1) | 5.8 (1) |
| Version B |  |  |  |  |  |  |
| List items |  |  |  |  |  |  |
| Length | 5.7 (.7) | 5.5 (.6) | 5.5 (.1) | 5.3 (.3) | 5.7 (.2) | 5.5 (.3) |
| Probe |  |  |  |  |  |  |
| Frequency | 55 (28) | 50 (53) | 29 (27) | 27 (41) | 51 (66) | 61 (94) |
| Length | 5.8 (.2) | 5.6 (2) | 4.5 (1) | 6 (1) | 5.8 (2) | 5.6 (1) |
|  |  |  |  |  |  |  |
| Legend: N-A/C = negative-associated plus combined; N-A = negative-associated; N-C = negative-combined; N-U = negative-unrelated; P-R = positive-related; P-U = positive-unrelated | | | | | | |

### Appendix 3 – Scoring rules for clustering and switching based on Troyer et al., 1997; see also Troyer & Moscovitch, 2006 in Poreh, 2006) copied verbatim and our own criterion (underlined).

For each protocol, six scores were calculated, including the total number of correct words generated, mean cluster size, and number of switches for phonemic and semantic fluency, respectively. These scores are defined as follows:

***Total number of correct words generated.*** This was calculated as the sum of all words produced, excluding errors and repetitions.

***Mean cluster size.*** Cluster size was counted starting with the second word in a cluster. That is, a single word was given a cluster size of 0, two words had a cluster size of 1, three words had a cluster size of 2, and so forth. Errors and repetitions were included. The mean cluster size was computed across the three phonemic trials and across the one or two semantic trials.

***Number of switches.*** This was calculated as the total number of transitions between clusters, including single words, for the three phonemic trials combined and for the one or two semantic trials combined. Errors and repetitions were not included.

Phonemic Fluency

Clusters on phonemic fluency trials consisted of successively generated words which shared any of the following phonemic characteristics:

***First letters***: words beginning with same first two letters, such as ‘‘arm’’ and ‘‘art’’

***Rhymes***: words that rhyme, such as ‘‘sand’’ and ‘‘stand’’

***First and last sounds***: words differing only by a vowel sound, regardless of the actual spelling, such as ‘‘sat,’’ ‘‘seat,’’ ‘‘soot,’’ ‘‘sight,’’ and ‘‘sought’’

***Homonyms***: words with two or more different spellings, such as ‘‘some’’ and ‘‘sum,’’ as indicated by the participant

Semantic Fluency

Clusters on semantic fluency trials consisted of successively generated words belonging to the same subcategories, as specified below. Commonly generated examples are listed for each subcategory, although listings are not exhaustive.

Animals

Living Environment

*Africa*: aardvark, antelope, buffalo, camel, chameleon, cheetah, chimpanzee, cobra, eland, elephant, gazelle, giraffe, gnu, gorilla, hippopotamus, hyena, impala, jackal, lemur, leopard, lion, manatee, mongoose, monkey, ostrich, panther, rhinoceros, tiger, wildebeest, warthog, zebra, meerkat

*Australian animals*: emu, kangaroo, kiwi, opossum, platypus, Tasmanian devil, wallaby, wombat

*Arctic/Far North animals*: auk, caribou, musk ox, penguin, polar bear, reindeer, seal.

*Farm animals*: chicken, cow, donkey, ferret, goat, horse, mule, pig, sheep, turkey, duck**,** owl

*North America animals*: badger, bear, beaver, bobcat, caribou, chipmunk, cougar, deer, elk, fox, moose, mountain lion, puma, rabbit, raccoon, skunk, squirrel, wolf

*Water animals*: alligator, auk, beaver, crocodile, dolphin, fish, frog, lobster, manatee, muskrat, newt, octopus, otter, oyster, penguin, platypus, salamander, sea lion, seal, shark, toad, turtle, whale

*Woodland*: badger, fox, hedgehog

Human Use

*Beasts of burden*: camel, donkey, horse, llama, ox

*Animals used for their fur*: beaver, chinchilla, fox, mink, rabbit

*Pets*: budgie, canary, cat, dog, gerbil, golden retriever, guinea pig, hamster, parrot, rabbit

Zoological Gardens

*Birds*: budgie, condor, eagle, finch, kiwi, macaw, parrot, parakeet, pelican, penguin, robin, toucan, woodpecker

*Bovine*: bison, buffalo, cow, musk ox, yak

*Canine*: coyote, dog, fox, hyena, jackal, wolf

*Deers*: antelope, caribou, eland, elk, gazelle, gnu, impala, moose, reindeer, wildebeest

*Feline*: bobcat, cat, cheetah, cougar, jaguar, leopard, lion, lynx, mountain lion, ocelot, panther, puma, tiger

*Fish*: bass, guppy, salmon, trout *Insects*: ant, beetle, cockroach, flea, fly, praying mantis

*Insectivores*: aardvark, anteater, hedgehog, mole, shrew

*Primates*: ape, baboon, chimpanzee, gibbon, gorilla, human, lemur, marmoset, monkey, orangutan, shrew

*Rabbits*: coney, hare, pika, rabbit

*Reptiles/Amphibians*: alligator, chameleon, crocodile, frog, gecko, iguana, lizard, newt, salamander, snake, toad, tortoise, turtle

*Rodents*: beaver, chinchilla, chipmunk, gerbil, gopher, groundhog, guinea pig, hamster, hedgehog,

marmot, mole, mouse, muskrat, porcupine, rat, squirrel, woodchuck

*Weasels*: badger, ferret, marten, mink, mongoose, otter, polecat, skunk

Supermarket Items

*Fruits*: applesauce, bananas, cranberries, juice, mango, nectarines, peaches, raisins *Vegetables*: avocado, beans, carrots, eggplant, olives, pickles, tomatoes, zucchini

*Dairy case items*: cheese, cream, cream cheese, eggs, milk, sour cream, yogurt

*Meats*: bacon, chicken, fish, hamburger, hot dogs, pork, salmon, sausage, tuna

*Beverages*: coffee, juice, lemonade, milk, orange juice, pop, tea, water, wine

*Condiments*: jelly, ketchup, marmalade, mayonnaise, pickles, relish, salad dressing

*Flavourings*: chives, cinnamon, parsley, pepper, sage, salt, vanilla, vinegar

*Sweets and snacks*: candy, cake, crackers, donuts, gum, ice cream, pie, popcorn, pudding, torte

*Grain products*: barley, bread, cereal, corn meal, flour, macaroni, meal, muffins, oats, rice

*Baking supplies*: baking powder, cornstarch, eggs, flour, salt, shortening, spices, vanilla

*Specific meals/dishes*: coffee, eggs, syrup, waffles; spaghetti, tomato sauce; lettuce, onions, radishes, salad dressing; pork, beans

*Household goods*: ammonia, bicarb, detergent, disinfectant, gift wrap, Kleenex, magazines, mop, pans, paper bags, paper towels, stamps, tin foil, toilet paper, washing powder, washing liquid, wax paper,

*Personal toiletries*: aspirin, comb, deodorant, medicine, mouthwash, toothpaste, vitamins

*Infrastructure*: aisles, basket, butcher, cash register, cashier, grocery bags, pharmacy, price tags, shelves, shopping cart, trolleys

*Essentials*: bread, butter, eggs, milk

*Sunday roast*: beef, chicken, gravy, lamb, meat, peas, pork, stock

*Electronics*: computers, earphones, headphones, laptops, phones, iPods, speakers

*Utensils*: fork, knives, peelers, rolling pin, spatula, spoon

*Clothes*: hats, socks

*Condiments*: brown sauce, ketchup, mayonnaise, Worcester sauce

Fruits

*Berries & Currants*: blackcurrants, blackberries, blueberries, gooseberries, strawberries, raspberries, redcurrants

*Bowl fruits*: apple, banana, pears, grapes, oranges, plums, peaches

*Exotic fruits*: kiwi, pineapple, tomato, mango, pomegranate, cherries, melon, peach, plums, nectarine, grape fruit, papaya, avocado, figs, apricots, quinoa, coconut

*Citric*: blood orange, grape fruit, kumquat, lemon, limes, nectarine, oranges, tangerine, satsumas

*Dried fruit*: dates, figs, prunes, raisins, currants

Musical Instruments

*Band*: guitar, drums, keyboard, bass

*Brass*: bugle, flute, French horn, horn, saxophone, trombone, trumpet, tuba,

*Percussion*: bass drum, bongo, castanets, chimes, cymbal, glockenspiel, gong, snare drum, spoons, tambourine, tamborim, triangle, drums, whistle, xylophone

*Wind* *instruments*: bassoon, clarinet, oboe, flute, recorder, saxophone

*String*: violin cello bass, banjo, double bass, guitar, harp, lyre, mandolin, ukulele

General Scoring Rules

In the case where two categories overlapped, with some items belonging to both categories, some items belonging exclusively to the first category, and some items belonging exclusively to the second category, the overlapping items were assigned to both categories. For example, for ‘‘dog, cat, tiger, lion,’’ the first two items were scored as pets, and the last three items were scored as feline. ‘‘Cat’’ was included in both the pet category and the feline category.

In the case where smaller clusters were embedded within larger ones, or two categories over- lapped, but all items could correctly be assigned to a single category, only the larger, common cate- gory was used. For example, for ‘‘sly, slit, slim, slam’’ all begin with ‘‘sl,’’ but an additional cluster was not scored for the last two words which differ only by a vowel sound.

### Appendix 4 – BIS/BAS scale given to participants and scoring criteria to participant responses taken from Carver and White (1994; <http://www.psy.miami.edu/faculty/ccarver/sclBISBAS.html>)

**BIS/BAS Scale**

Each item of this questionnaire is a statement that a person may either agree with or disagree with.  For each item, indicate how much you agree or disagree with what the item says.  Please respond to all the items; do not leave any blank.  Choose only one response to each statement.  Please be as accurate and honest as you can be.  Respond to each item as if it were the only item.  That is, don't worry about being "consistent" in your responses.  Choose from the following four response options:

  1 = very true for me

  2 = somewhat true for me

  3 = somewhat false for me

  4 = very false for me

1.  A person's family is the most important thing in life.

2.  Even if something bad is about to happen to me, I rarely experience fear or nervousness.

3.  I go out of my way to get things I want.

4.  When I'm doing well at something I love to keep at it.

5.  I'm always willing to try something new if I think it will be fun.

6.  How I dress is important to me.

7.  When I get something I want, I feel excited and energized.

8.  Criticism or scolding hurts me quite a bit.

9.  When I want something I usually go all-out to get it.

10.  I will often do things for no other reason than that they might be fun.

11.  It's hard for me to find the time to do things such as get a haircut.

12.  If I see a chance to get something I want I move on it right away.

13.  I feel pretty worried or upset when I think or know somebody is angry at me.

14.  When I see an opportunity for something I like I get excited right away.

15.  I often act on the spur of the moment.

16.  If I think something unpleasant is going to happen I usually get pretty "worked up."

17.  I often wonder why people act the way they do.

18.  When good things happen to me, it affects me strongly.

19.  I feel worried when I think I have done poorly at something important.

20.  I crave excitement and new sensations.

21.  When I go after something I use a "no holds barred" approach.

22.  I have very few fears compared to my friends.

23.  It would excite me to win a contest.

24.  I worry about making mistakes.

**Scoring Criteria**

Items other than 2 and 22 are reverse-scored.

BAS Drive:  3, 9, 12, 21

BAS Fun Seeking:  5, 10, 15, 20

BAS Reward Responsiveness:  4, 7, 14, 18, 23

BIS:  2, 8, 13, 16, 19, 22, 24

Items 1, 6, 11, 17, are fillers.
